# Improved hippocampal dose with reduced margin radiotherapy for glioblastoma multiforme

**DOI:** 10.1186/1748-717X-9-20

**Published:** 2014-01-10

**Authors:** Arif N Ali, Tomi Ogunleye, Claire W Hardy, Hui-Kuo Shu, Walter J Curran, Ian R Crocker

**Affiliations:** 1 Department of Radiation Oncology, Emory University, 1365 Clifton Rd. NE, Atlanta, GA 30322, USA; 2 Winship Cancer Institute, Emory University, 1365 Clifton Rd. NE, Atlanta, GA 30322, USA

**Keywords:** Glioblastoma multiforme, GBM, Hippocampus

## Abstract

**Background:**

To dosimetrically evaluate the effect of reduced margin radiotherapy on hippocampal dose for glioblastoma multiforme (GBM) patients.

**Methods:**

GBM patients enrolled on the Radiation Therapy Oncology Group (RTOG) 0825 trial at our institution were identified. Standard RTOG 0825 expansions were 2 cm + 3-5 mm from the gross tumor volume (GTV) to the clinical tumor volume (CTV) and from the CTV to the planning tumor volume (PTV), respectively. These same patients also had reduced margin tumor volumes generated with 8 mm (GTV to CTV) + 3 mm (CTV to PTV) expansions. Individual plans were created for both standard and reduced margin structures. The dose-volume histograms were statistically compared with a paired, two-tailed Student’s t-test with a significance level of p < 0.05.

**Results:**

A total of 16 patients were enrolled on RTOG 0825. The reduced margins resulted in statistically significant reductions in hippocampal dose at all evaluated endpoints. The hippocampal D_max_ was reduced from a mean of 61.4 Gy to 56.1 Gy (8.7%), D_40%_ was reduced from 49.9 Gy to 36.5 Gy (26.9%), D_60%_ was reduced from 32.7 Gy to 18.7 Gy (42.9%) and the D_80%_ was reduced from 27.3 Gy to 15.3 Gy (44%).

**Conclusions:**

The use of reduced margin PTV expansions in the treatment of GBM patients results in significant reductions in hippocampal dose. Though the exact clinical benefit of this reduction is currently unclear, this study does provide support for a future prospective trial evaluating the neurocognitive benefits of reduced margin tumor volumes in the treatment of GBM patients.

## Background

Glioblastoma Multiforme (GBM) has traditionally been a devastating disease with median life expectancies of a year or less from the time of diagnosis [1]. In recent years, however, there have been several significant advances in the treatment and stratification of patients with GBM that have resulted in life expectancies in excess of 21 months for select cohorts [2]. Specifically, the combination of temozolomide and radiotherapy has increased the median survival from 12.1 months for radiotherapy alone to 14.6 months with temozolomide and radiotherapy [3]. More striking is the long-term follow-up data for temozolomide and radiotherapy, which reveals that 9.8% of patients treated with concurrent therapy were alive at 5 years [4]. This point is further magnified when methyl-guanine methyl transferase gene (MGMT) methylation, which has been previously shown to be an independent prognostic indicator and predictor of benefit from temozolomide, is considered [2,5-8]. In those patients with MGMT methylation treated with a combination of temozolomide and radiotherapy the 5-year overall survival (OS) was shown to be as high as 13.8% [4].

Given the increased life expectancy of GBM patients in general and specifically those with MGMT methylation, attention has begun to focus on quality of life issues in addition to survival [9,10]. Multiple studies have demonstrated memory, learning, and other neurocognitive deficits in long-term survivors of GBM and other high grade gliomas previously treated with radiotherapy [6,9-17]. The retrospective review by Imperato et al. found that 2 out of 5 long-term high grade glioma survivors had significantly impaired short-term memory function [13]. Likewise, Lieberman et al. found that 2 out of 3 long-term astrocytoma survivors had diffuse cortical dysfunction so disabling that the patients could no longer work [14]. Perhaps the largest series was that by Peters et al. that found that out of 123 long-term GBM survivors with a median survival of 74.5 months, greater than 25% required the use of some psychoactive medications, thus indicating significant neurocognitive sequela [10]. It seems that the decreased neurocognitive performance of post-radiation glioma patients may be due, in large part, to memory dysfunction. Salander et al. evaluated 30 patients with malignant gliomas that had received radiation and found no clear impairment in global intellectual abilities or visual imagery, but did find a profound deficit in long-term memory [15].

It has been long known that the hippocampus is primarily involved with learning and long-term memory formation [18-23]. In primate studies, it was found that monkeys with bilateral removal of the hippocampus and amygdala had significantly impaired spatial learning and memory [24-26]. In patients, isolated lesions of the hippocampus have been consistently associated with anterograde amnesia and learning dysfunction [27-30].

Given the association of hippocampal lesions with memory dysfunction and the significant decline in neurocognitive performance after cranial irradiation, a study was performed that investigated the association of hippocampal radiation dose with neurocognitive impairment [31]. Gondi et al. found that a dose greater than 7.3 Gy to 40% of the bilateral hippocampus was associated with long-term impairment in Wechsler Memory Scale-Word List delayed recall for fractionated stereotactic radiotherapy treatment of benign or low-grade adult brain tumors [31]. With these results, there has recently been significant interest in minimizing dose to the hippocampus during whole brain radiation for the palliation of brain metastases and prophylactic cranial irradiation [32-35]. To date, however, there have been no explorations of hippocampal sparing in the setting of GBM even though neurocognitive decline appears to be just as significant after radiation for GBM and the life expectancy for a sizeable cohort of GBM patients is now the same or better than that for patients with brain metastases.

At our institution, we have previously reported the use of reduced margin radiotherapy in the management of GBM patients [36]. McDonald et al. found that use of total PTV margins of less than 1 cm resulted in radiographic tumor progression rates comparable with standard 2.3 cm to 2.5 cm GBM PTV margins. Specifically, 93% of the failures were in-field, further indicating that there was likely no recurrence detriment of a reduced PTV margin [36]. The purpose of the current study is to dosimetrically evaluate the effect of reduced margin radiotherapy for GBM patients on hippocampal dose compared with standard margin radiotherapy and determine if there is likely to be a clinical benefit in terms of improved neurologic function based on previously published hippocampal radiation/neurocognitive toxicity dose thresholds [31].

## Methods

### Patients

Patients treated for glioblastoma multiforme at our institution and enrolled on the Radiation Therapy Oncology Group (RTOG) trial 0825 were identified. RTOG 0825 was a phase III trial of conventional concurrent chemoradiation and adjuvant temozolomide plus bevacizumab versus conventional concurrent chemoradiation and adjuvant temozolomide alone in patients with newly diagnosed glioblastoma [37]. A total of 16 patients, treated post-operatively according to RTOG 0825 were included in this analysis.

### Simulation and planning

CT simulation scans were acquired with patients in the supine position using a thermoplastic head mask for immobilization and 3 mm CT slice thickness. Additionally, post-operative magnetic resonance images (MRI) were used for treatment planning purposes. The CT simulation image was registered to the MRI with the use of Velocity AI (Velocity Medical Solutions, Atlanta, GA) or Brainlab (Brainlab Inc., Westchester, IL) software. RTOG 0825 required both an initial planning target volume (PTV1) treated to 46 Gy in 23 fractions and a boost planning target volume (PTV2) treated to an additional 14 Gy in 7 fractions. In accordance with RTOG 0825 specifications, the T2 or FLAIR abnormality was expanded by a 2 cm margin to create the CTV1 and the T1 contrast-enhanced volume was expanded by 2 cm to create CTV2. Both CTV’s were expanded by an additional 5 mm to create the standard margin PTV1 and PTV2. Normal structures (and dose constraints) required by RTOG 0825 were the lenses (D_max_ = 7 Gy), retinae (D_max_ = 50 Gy), optic nerves (D_max_ = 55 Gy), optic chiasm (D_max_ = 56 Gy) and brainstem (D_max_ = 60 Gy). The left, right, and bilateral hippocampus were retrospectively contoured on the T1 post-contrast MRI and imported into the treatment planning software, Eclipse (Varian, Palo Alto, CA). Additionally, reduced margin CTV1 and CTV2 structures were created by expanding the FLAIR and T1 contrast-enhanced volume by 8 mm, respectively. The reduced margin PTV1 and PTV2 were created by expanding the CTV’s by an additional 3 mm. Both the standard margin and reduced margin PTV’s were planned using a sliding window IMRT technique with the RTOG normal tissue and target constraints of less than 10% inhomogeneity within the target volume and at least 95% of the PTV receiving 100% of the prescribed dose.

### Statistics and analysis

The primary means of comparing plans were dose-volume histograms (DVH). The appropriate dose and volume parameters for OAR were defined and used for comparison. Additionally, the V_95%_ and V_98%_ for PTV2 was used to assess target coverage. These parameters were statistically compared with a paired, two-tailed Student’s t-test with a significance level of p < 0.05.

## Results

Table 1 contains various PTV dosimetric parameters for both the standard margin and reduced margin plans. It is seen that volume receiving 95% of the prescribed dose (V_95%_) is above 99% of the PTV for both the standard and reduced margin plans. It would be expected that there should be equal coverage of the PTV between the standard margin and reduced margin plans as target coverage is the effect of the equal PTV constraints imposed by the operator.

**Table 1:** Planning tumor volume (PTV2) dosimetric parameters

	**Standard margin**		**Reduced margin**				
**Parameter**	**Mean**	**St. Dev.**	**Mean**	**St. Dev.**	**Difference**	**(%)**	**p-value**
Max dose (Gy)	63.59	0.96	63.60	1.13	−0.006	−0.01%	0.982
Mean dose (Gy)	61.92	0.60	61.94	1.02	−0.018	−0.03%	0.934
V98% dose (%)	99.47	0.70	99.47	1.36	0.006	0.01%	0.982
V95% dose (%)	99.81	0.37	99.72	0.81	0.089	0.09%	0.596

**Figure 1:**
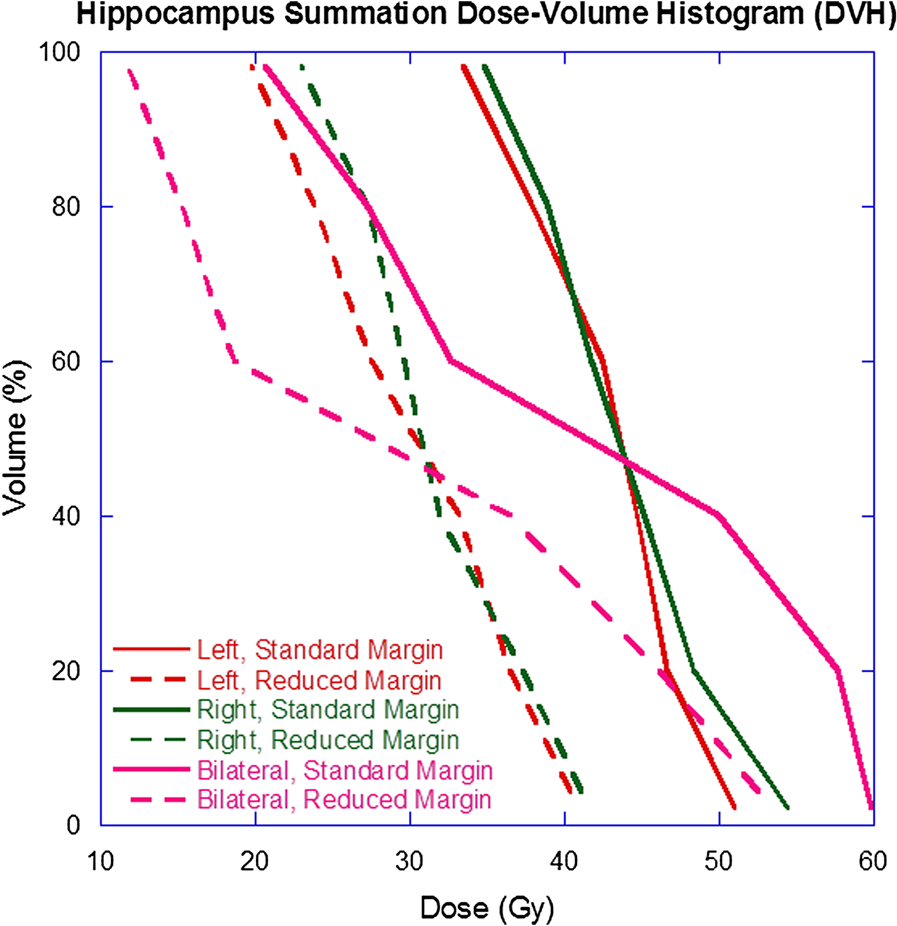
Summation dose-volume histogram (DVH) for the left, right, and bilateral hippocampal structures comparing standard and reduced margins.

Though the standard margin and reduced margin PTV’s received similar dose, it is seen from Table 2 that the reduced margin volumes translated to statistically significant reductions in all bilateral hippocampal dosimetric parameters. The reduction in dose varied from 5.3 Gy (8.7%) for the maximum dose to 12.0 Gy (44.0%) for the D_80%_ with a 17.2 Gy (38.2%) reduction in the median dose. Additionally, as seen in Figure 1, there are similar reductions in dose with the reduced margin PTV’s when the bilateral hippocampal structure is divided into individual left and right hippocampal structures.

**Table 2:** Bilateral hippocampal dosimetric parameters

	**Standard margin**		**Reduced margin**				
**Parameter**	**Mean**	**St. Dev.**	**Mean**	**St. Dev.**	**Difference (Gy)**	**(%)**	**p-value**
Max dose (Gy)	61.4	1.4	56.1	8.6	5.3	8.7%	0.014
Mean dose (Gy)	41.9	9.1	29.9	11.6	12.0	28.7%	<0.001
Median dose (Gy)	45.2	17.2	27.9	15.6	17.2	38.2%	<0.001
D2% (Gy)	59.9	5.1	53.4	12.1	6.4	10.7%	0.008
D20% (Gy)	57.6	8.5	46.1	16.8	11.5	20.0%	0.001
D40% (Gy)	49.9	14.9	36.5	19.6	13.4	26.9%	<0.001
D60% (Gy)	32.7	13.5	18.7	10.9	14.0	42.9%	<0.001
D80% (Gy)	27.3	12.1	15.3	10.0	12.0	44.0%	<0.001
D98% (Gy)	20.7	9.3	11.8	7.7	8.9	42.9%	<0.001

## Discussion

The current study confirms a statistically significant dosimetric reduction in hippocampal dose with the use of reduced margin radiotherapy. Specifically, it has been determined that the hippocampal dose reduction varies between 8.7% to 44.0% depending on the dosimetric parameter considered. Though a dose reduction of up to 44% to the hippocampus is quite significant in magnitude, it remains unclear whether there exists any translatable improvements in clinical memory function or other neurocognitive endpoints. The most recent study to explore the link between hippocampus dose and neurocognitive function is that by Gondi et al. [31] This study involved 29 patients with benign or low-grade brain tumors treated with fractionated stereotactic radiotherapy. Gondi et al. converted all doses to 2 Gy per fraction biologically equivalent doses and evaluated neurocognitive function with the Wechsler Memory Scale-III Word List. They found that a 2 Gy per fraction equivalent dose (EQD_2_) to 40% of the bilateral hippocampi greater than 7.3 Gy was associated with impaired recall function. Additionally, they found a significant dose–response relationship with an of 14.9 Gy to the bilateral hippocampi and a slope of 0.540. Though the hippocampal D_40%_ doses reported in the current study for both the standard margin PTV (49.9 Gy) and the reduced margin PTV (36.5 Gy) are above the 7.3 Gy threshold, this represents an approximately 13.4 Gy (26.9%) reduction in the hippocampal D_40%_ dose. Assuming that the dose–response relationship observed by Gondi et al. continued to be linear with a slope of 0.540, a 26.9% reduction in hippocampal D_40%_ dose should translate to a 14.5% improvement on the Wechsler Memory Scale-III Word Lists Delayed Recall at 18 months. In addition to Gondi et al., another group has reported a dose–response relationship for the radiation of the hippocampus in low grade or anaplastic gliomas [38]. Of course, with significantly limited cohort sizes and follow-up, the quantitative relationship between hippocampal dose and memory function reported by Gondi et al. and Mahajan et al. should be applied with an excess of caution and care [31,38]. However, the reduction of hippocampal dose with the use of reduced margins in GBM patients in the context of a potential dose–response relationship is still encouraging.

Factors that could potentially confound the previously observed relationship between hippocampal dose and neurocognitive effects would be secondary effects of systemic agents as well as direct neurocognitive effects of disease progression [39]. Hahn et al. evaluated patients that had not received any prior radiotherapy and found worse cognitive function in patients with left sided lesions and higher grade gliomas (GBM) [40]. This would indicate that, at baseline, there is some direct cognitive impairment from the intracranial disease. In fact, mental status on presentation has been found to be one of the most significant predictors of prognosis for malignant gliomas [41]. Additionally, it has been demonstrated that GBM patients with tumor progression had more significant cognitive decline than patients with stable disease, though it was unclear whether this was due to disease progression or anti-epileptic use [42]. Anti-epileptic drug use has been previously implicated in the reduction of most cognitive functions except memory in a recent study on low-grade glioma patients [43]. Conversely, corticosteroids have been demonstrated to directly result in a decline in declarative and working memory [44]. Additionally, corticosteroids have been found to result in decreased hippocampal activity and blood flow [44].

While there are not yet enough data to fully dissect the specific contribution of radiation therapy to cognitive decline compared to systemic agents and disease progression, there is sufficient evidence to indicate that it is a significant factor. As mentioned previously, there is currently significant interest in mitigating radiation-induced effects upon the hippocampus as a means to improve memory and other neurocognitive outcomes in long-term survivors with metastatic brain disease. The primary means of reducing radiation dose to the hippocampus has been to selectively avoid that structure with the use of IMRT. This is a reasonable approach for brain metastases as there is published evidence indicating that only 8.6% of patients have perihippocampal brain metastases and only 3.0% of brain metastases are perihippocampal [45]. In the current RTOG 0933 protocol studying hippocampal avoidance during whole brain radiation, the perihippocampal region is defined as the hippocampi with a 5 mm margin [46]. In the current RTOG 0825 protocol studying the use of bevacizumab in GBM patients, the PTV is defined as the GTV with a 2.3 cm to 2.5 cm margin. With such a large margin on the GTV, it would be difficult to selectively avoid the hippocampus in a majority of GBM lesions [37]. Using only the McDonald et al. reduced GBM margin of 1.2 cm from the GTV to the PTV (without selective hippocampal avoidance), this study has demonstrated up to a 44% reduction in dose to the hippocampus compared to standard RTOG margins. Additional hippocampal dose reduction may be expected with selective hippocampal avoidance in GBM patients. Though there is not yet an active hippocampal sparing protocol for GBM patients, the reduced margins from MacDonald et al. should safely enable hippocampal sparing in a greater proportion of GBM patients than standard margins [36].

A trial could conceivably be conducted to evaluate and quantify the neurocognitive benefits of reduced margin radiation therapy for GBM patients. Such a trial would likely require patients to be randomized between a standard margin arm (2.3 cm to 2.5 cm) and a reduced margin arm. Both arms would then receive identical Temodar treatment. The primary evaluated outcome would be neurocognitive function and the secondary outcomes would include radiographic progression, overall survival, and quality of life assessments. The hippocampus would be contoured as part of the trial and hippocampal dosimetric data from the treatment plan (D_max_, D_40%_, etc.) could then be correlated with late neurocognitive effects to generate further insight into the dose–response relationship of the hippocampal organ.

## Conclusions

This study builds upon previously published data demonstrating the safety and efficacy of reduced PTV margins for GBM patients by dosimetrical evaluating and quantifying the effect of the reduced margins on hippocampal dose. It was found that a hippocampal dose reduction of 8.7% to 44% may be realized with the reduced PTV margins compared to standard RTOG 0825 margins for GBM patients. It is anticipated the reduced PTV margins will also allow selective hippocampal sparing in a greater proportion of GBM patients compared to standard margins. While the exact clinical benefit of this dose reduction is currently unclear, this study does provide support for a prospective clinical trial investigating the neurocognitive benefits of reduced margin radiotherapy and hippocampal sparing for GBM patients.

## Competing interests

The authors declare that they have no competing interests.

## Authors’ contributions

AA: Organized and supervised the collection of data, performed the data statistical analysis, contoured the hippocampal structures, created all of the figures, and drafted the manuscript. TO: Performed the dosimetric calculations and generated the dosimetric plans. CH: Helped to collect and organize the data from the planning software to the spreadsheet for analysis. HS: Helped to draft and revise the manuscript. WC: Helped to draft and revise the manuscript. IC: Conceived of the study, participated in its design, and helped draft and revise the manuscript. All authors read and approved the final manuscript.
